# A network pharmacology study of mechanism and efficacy of Jiawei Huanglian-Wendan decoction in polycystic ovary syndrome with insulin resistance

**DOI:** 10.1097/MD.0000000000032057

**Published:** 2022-12-02

**Authors:** Na Shi, Yuhe Zhou, Hongbo Ma

**Affiliations:** a The First Clinical Medical College, Shandong University of Traditional Chinese Medicine, Jinan, China; b Department of Traditional Chinese Medicine, Shandong Provincial Hospital Affiliated to Shandong First Medical University, Jinan, China.

**Keywords:** IR, network pharmacology, PCOS, traditional Chinese medicine

## Abstract

Polycystic ovary syndrome (PCOS) is a common reproductive metabolic disorder, normally accompanied by insulin resistance (IR). The specific pathogenesis of this disease remains unclear. To identify the underlying pathogenesis of PCOS with IR and explore the potential efficacy and mechanism of Jiawei Huanglian-Wendan decoction (JHWD) by a network pharmacology approach. The effective components and the potential drug and disease-related targets are retrieved. Drug-disease overlapped targets are being obtained by Venny analysis. The construction of protein-protein interaction network relied on Search Tool for the Retrieval of Interacting Genes/Proteins database (STRING), after uploading drug-disease overlapped targets. The drug-component-target-disease interaction network map was displayed , after importing their data into Cytoscape 3.7.2 software. Bioinformatics analyses are being performed by Metascape and Kyoto Encyclopedia of Genes and Genomes databases, respectively. Further, molecular docking analysis was carried out using AutoDock software. Finally, the influence of JHWD is verified by means of traditional Chinese medicine syndrome score, the rate of resumption of normal menstrual cycles and regular ovulation, the blood lipid levels, the blood glucose and insulin levels, and the inflammatory cytokines in PCOS with IR patients. Four primary interaction networks of JHWD are constructed. The enrichment analysis of PCOS-IR-related targets demonstrated that the top enriched pathways in the development of PCOS with IR are pathways in cancer, metabolic, phosphoinositide-3-kinase-protein kinase B signaling, lipid and atherosclerosis, and mitogen-activated protein kinase signaling pathways. Molecular docking analysis revealed strong binding interactions of the key targets with the active components. Further confirmations showed that the active components of JHWD exhibited significant clinical efficacy in improving the clinical syndromes, menstrual cyclicity and ovulatory function, and significantly reducing the blood lipid levels, blood glucose and insulin levels, and inflammatory cytokines in PCOS with IR patients. The combination of the network pharmacological analysis and clinical validation stated that the active compounds in JHWD could regulate glycolipid metabolism, reduce IR, and exert anti-inflammatory effects in the treatment of PCOS with IR, promoting Chinese classical formulations.

## 1. Introduction

Polycystic ovary syndrome (PCOS), a heterogeneous disease marked by insulin resistance (IR), has become a challenging pathological condition in gynecology and endocrinology due to unclear pathogenesis. In this context, chronic low-grade inflammation has been implicated as a key pathogenic mechanism of PCOS and consequent IR.^[1]^ IR can further aggravate chronic inflammation by activating the inflammatory response pathway. Therefore, ameliorating low grade chronic inflammation may be an effective therapy for PCOS with IR. However, the therapeutic options for PCOS with IR are minimal, as most of these drugs result in severe adverse effects and unclear long-term risk-benefit ratio.

To this end, traditional Chinese medicine (TCM) offers unique advantages in clinical practice via the synergism of multiple targets, links, and pathways as well as fewer side effects compared to chemotherapeutic agents,^[2]^ significantly improving the holistic functional status of patients. From the perspective of TCM, phlegm-dampness stagnation is the primary etiology and pathology of PCOS with IR, making disorder of qi movement of the body, disharmony of qi and blood, as well as stagnation of skin and meridian. Consequently, these pathological conditions lead to the typical symptoms of PCOS, such as menstrual irregularity, infertility, obesity, hirsutism, and acne.

Huanglian-Wendan decoction is a classic traditional Chinese prescription recorded in *liu yin tiao bian* written by Lu Tingzhen in the Qing Dynasty. Studies confirmed that Huanglian-Wendan decoction could improve liver lipid accumulation and enhance insulin sensitivity in IR rats.^[3]^ Moreover, the Huanglian-Wendan decoction could decrease the area of aortic plaques and prevent the progression of atherosclerosis.^[4]^ It was demonstrated that Huanglian-Wendan decoction could ameliorate dysregulated glucose and lipid metabolism and regulate pancreatic β-cells.^[5]^ In this framework, Jiawei Huanglian-Wendan decoction (JHWD), a modified compound prescription, is often used to clear heat and dry dampness, resolve phlegm, regulate qi, disperse stasis, obviate retention, strengthen spleen, and replenish kidney. Notably, the JHWD is comprised of several TCM molecules: Coptidis Rhizoma (Huanglian), Scutellariae Radix (Huangqin), Caulis Bambusae in Taeniam (Zhuru), Aurantii Fructus Immaturus (Zhishi), Poria Cocos (Schw.) Wolf. (Fuling), Citrus Reticulata (Chenpi), Cyperi Rhizoma (Xiangfu), Arum Ternatum Thunb (Banxia), Radix Salviae (Danshen), Angelicae Sinensis Radix (Danggui), Cuscutae Semen (Tusizi), and Atractylodes Macrocephala Koidz. (Baizhu). Our previous studies have confirmed that JHWD had achieved high efficacy in treating PCOS with IR.^[6,7]^ However, the potential mechanism of JHWD for PCOS with IR is not well defined.

**Figure 1.**
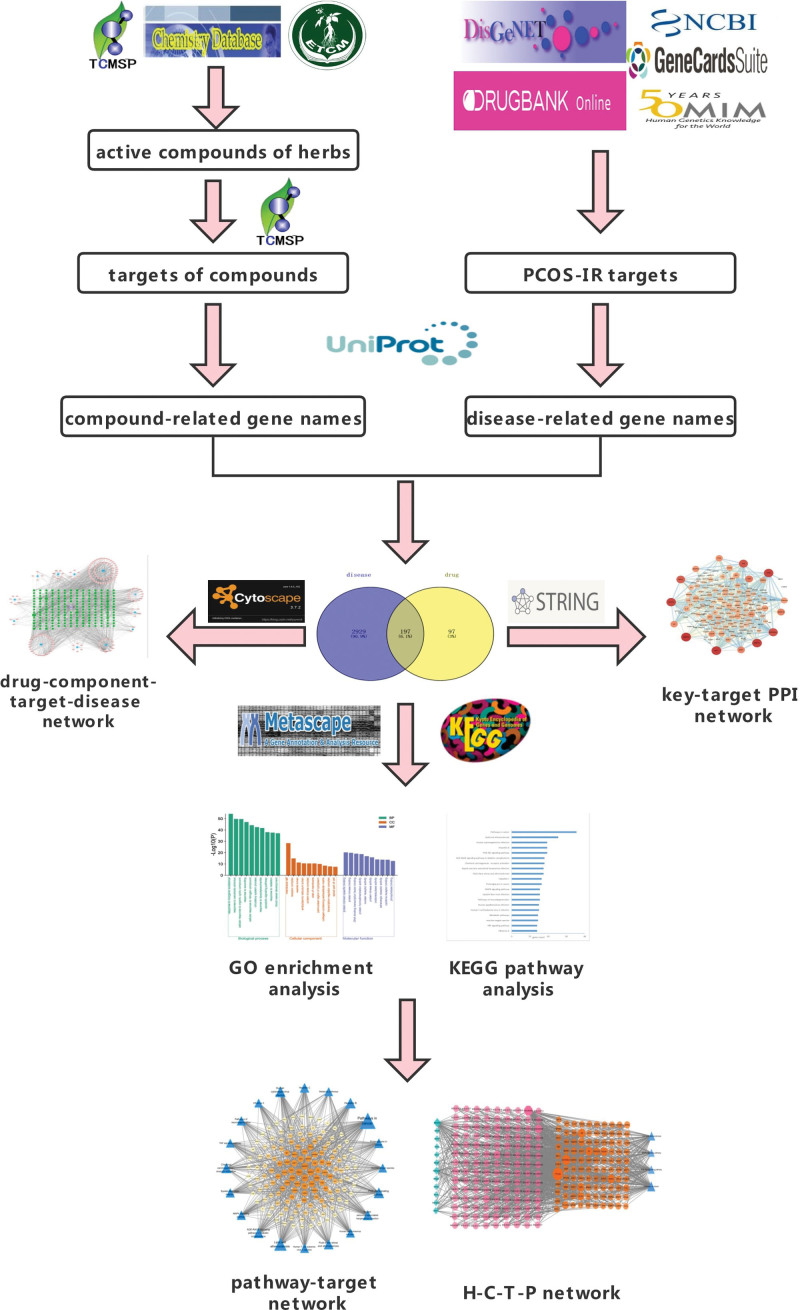
The flowchart of the network pharmacology study.

The network pharmacology approach, a new approach, is often employed to uncover the pathogenesis of diseases and analyze the drug’s mechanisms of action through various networks of multi-level interactions with a perspective of TCM. It provides a valuable basis to exploring the drug’s mechanism of action, bridging the gap between TCM and chemotherapeutics. However, these predictions by network pharmacology approach need experimental validation, relying on the clinical experiments.^[8]^ Therefore, this study aims to identify the underlying pathogenesis of PCOS with IR and the potential mechanistic action of JHWD in treating PCOS with IR using the network pharmacology method. Further, a clinical experiment is performed to verify the efficacy of JHWD in treating PCOS with IR and explore insight into the underlying mechanisms (Fig. 1).

## 2. Materials and Methods

### 2.1. Network pharmacology

#### 2.1.1. Obtaining the targets of active components.

The components of 12 herbs in JHWD were acquired from the TCM System Pharmacology database (TCMSP, https://tcmspw.com/index.php),^[9]^ Chemistry database (http://www.organchem.csdb.cn/),^[10]^ and the Encyclopedia of Traditional Chinese Medicine (ETCM, http://www.tcmip.cn/ETCM/index.php/Home/),^[11]^ using the Chinese pinyin “Huanglian, Huangqin, Zhuru, Zhishi, Fuling, Chenpi, Xiangfu, Banxia, Danshen, Danggui, Tusizi, and Baizhu” as keywords. Further, we screened eligible components based on ADME (absorption, distribution, metabolism, and excretion).^[12]^ Then, in this study, we identified active components under standard conditions of oral bioavailability (OB) of ≥30% and drug-likeness (DL) of ≥0.18.^[13]^ The target names of each herb were screened from the TCMSP database.

#### 2.1.2. Acquisition of drug-related and disease-related targets.

With “polycystic ovary syndrome” “insulin resistance” as keywords, disease-related targets were searched and screened from the following 5 existing databases: (1) GeneCards (https://www.genecards.org/),^[14]^ (2) Drug Bank (https://www.drugbank.ca/),^[15]^ (3) National Center for Biotechnology Information (NCBI, https://www.ncbi.nlm.nih.gov/), (4) Disgenet (https:www.genecards.org/),^[16]^ and (5) Online Mendelian Inheritance in Man (OMIM, http://www.omim.org).^[17]^ The search results from each database were combined, and duplicates were eliminated. Then, we converted the drug-related and disease-related target protein names into gene names by the UniProt Knowledgebase (UniProtKB, https://www.uniprot.org/),^[18]^ with the species limited to “Homo sapiens.”

#### 2.1.3. Gathering drug-disease overlapped targets.

The collected drug-related and disease-related targets were input into Venny 2.1 (https://bioinfogp.cnb.csic.es/tools/venny/), and the overlapped targets between drug and disease were gained using Venny online platform.

#### 2.1.4. Methods for network construction.

To illustrate the potential mechanisms of JHWD in PCOS with IR treatment, some major networks were constructed, including key-target protein-protein interaction (PPI) network, drug-component-target-disease network, and target-pathway network, using Search Tool for the Retrieval of Interacting Genes/Proteins (STRING) database (https://string-db.org/) and Cytoscape 3.7.2 software.

#### 2.1.5. Bioinformatics analyses.

Gene Ontology (GO) and Kyoto Encyclopedia of Genes and Genomes (KEGG) pathway enrichment analysis were performed using Metascape database (https://metascape.org/)^[19]^ and KEGG database (https://www.kegg.jp/).^[20]^ GO categories included various criteria of biological process (BP), cellular component (CC), and molecular function (MF). Further, the enrichment analysis results were screened, using the screening criteria of *P* < .01.

#### 2.1.6. Module analysis.

The 2-dimensional (2D) ligand structures of active components were downloaded from PubChem (https://pubchem.ncbi.nlm.nih.gov) in .sdf format. The 3-dimensional (3D) structures of key target proteins were downloaded from RCSB PDB database (https://www.rcsb.org/) in .pdb format. Then, the 2-dimensional (2D) ligands were optimized using ChemBio 3-dimensional (3D) software (version 20.0) in .mol2 format. AutoDock Tools (ADT version 1.5.7) was used for protein and ligand preparation, and AutoDock Vina (version 1.1.2) was used for molecular docking. Finally, the further visualization of the docking results was performed in PYMOL software (version 2.5.4).

### 2.2. Clinical study

#### 2.2.1. Patients.

From April 2019 to June 2020, PCOS with IR patients (n = 80, aged 20–43) who were treated at Shandong Provincial Hospital were randomly distributed into 2 groups: experimental and control groups, in which each group was allocated with 40 subjects.

#### 2.2.2. Inclusion criteria.

1. The clinical diagnosis of PCOS was performed by following the Chinese Guidelines for PCOS based on revised Rotterdam Consensus criteria.^[21]^
2. The homeostasis model assessment of insulin resistance (HOMA-IR) value of ≥1.66 was investigated for IR.^[22]^
3. TCM syndrome elements diagnosis standard: phlegm-dampness syndrome refers to the Guidelines for Diagnosis and Treatment of Common Diseases of Gynecology in Traditional Chinese Medicine.^[23]^

#### 2.2.3. Exclusion criteria were listed as follows.

Patients with conditions of diabetes mellitus, hyperprolactinemia, hypothyroidism, hyperthyroidism, non-classic congenital adrenal hyperplasia, Cushing syndrome, pelvic organ pathologies, severe hepatic or renal disease, malignant tumors, psychosis, and digestive tract disease; patients on antibiotics, or other medications affecting the sex hormones in the past 3 months; and patients with hypersensitivity to metformin.

#### 2.2.4. Type of interventions.

All the patients were received with metformin (500 mg thrice a day) and Diane-35 (1 tablet a day from days 2–5 of the menstrual cycle, for 21 days consecutively) for 3 menstrual cycles.

Moreover, apart from the control group, the experimental group was provided with JHWD deprived from the following herbs: 6g Coptidis Rhizoma, 10g Scutellariae Radix, 15g Caulis Bambusae in Taeniam, 12g Aurantii Fructus Immaturus, 15g Poria Cocos (Schw.) Wolf., 12g Citrus Reticulata, 12g Cyperi Rhizoma, 10g Arum Ternatum Thunb, 12g Radix Salviae, 12g Angelicae Sinensis Radix, 24g Cuscutae Semen, and 15g Atractylodes Macrocephala Koidz.. These Chinese herbal pieces for decoction were provided by TCM pharmacy of Shandong Provincial Hospital, Affiliated to Shandong First Medical University. Herbs were boiled in water for 30 minutes and condensed into 300mL decoction. Patients were administrated with 150mL twice a day after meal for 3 menstrual cycles.

The Ethics Committee of the Shandong Provincial Hospital Affiliated to Shandong First Medical University had approved the designed study protocol (BF201902011-02). In accordance with the guidelines, all the subjects who participated in the study have signed the written informed consent prior to conducting the study.

#### 2.2.5. Outcome measures.

1. Phlegm-dampness syndrome score: The clinical efficacy is determined by symptomatic improvement upon treatment, according to the Guiding Principles for Clinical Research of New Chinese Medicines (Ministry of Health of the People’s Republic of China, 2002).
2. The rate of resumption of normal menstrual cycles and regular ovulation.
3. Blood sample collection and testing. All blood samples were collected after 8 hours of fasting. Initially, the fasting blood glucose (FBG) and the fasting insulin (FINS) were recorded to enumerate the insulin resistance index (HOMA-IRI = (FBG × FINS)/22.5). Further, the levels of tumor necrosis factor-alpha (TNF-α), interleukin-1 (IL-1), and interleukin-6 (IL-6) in serum were measured by flow cytometry (BD FACSCanto II, BD Biosciences). Finally, the concentrations of triglycerides (TG), total cholesterol (TC), and low-density lipoprotein cholesterol (LDL-C) were enumerated using an automatic biochemical analyzer (AU5800, Beckman Coulter).

#### 2.2.6. Statistics analysis.

The statistical analyses were performed using the SPSS software 20.0. Data were expressed as mean ± standard deviation (x ± s). The measured data conforming to a normal distribution were analyzed using the *t* test for comparisons between 2 groups; otherwise, the nonparametric test was performed. The counting data were presented as numbers or percentages, and comparison between groups was conducted by χ^2^ test. *P* < .05 was considered as statistically significant, and *P* < .01 as statistically highly significant.

## 3. Results

### 3.1. Network pharmacology approach

#### 3.1.1. Active components of JHWD.

A total of 180 active components were collected, including 11 from Coptidis Rhizoma, 32 from Scutellariae Radix, 12 from Arum Ternatum Thunb., 4 from Atractylodes Macrocephala Koidz., 5 from Citrus Reticulata, 2 from Angelicae Sinensis Radix, 59 from Radix Salviae, 6 from Poria Cocos (Schw.) Wolf., 10 from Cuscutae Semen, 16 from Cyperi Rhizoma, 19 from Aurantii Fructus Immaturus, and 4 from Caulis Bambusae in Taeniam. The active components of Coptidis Rhizoma, the main herb of JHWD, are presented in Table 1.

**Table 1:** Active components of coptidis rhizoma.

Mol ID	Molecule name	OB (%)	DL
MOL001454	Berberine	36.86125	0.77665
MOL002894	Berberrubine	35.73551	0.7269
MOL002897	Epiberberine	43.09233	0.7761
MOL002903	(R)-Canadine	55.36687	0.77465
MOL002904	Berlambine	36.6809	0.81596
MOL002907	Corchoroside A_qt	104.9542	0.77599
MOL000622	Magnograndiolide	63.70888	0.18833
MOL000785	Palmatine	64.60111	0.64524
MOL000098	Quercetin	46.43335	0.27525
MOL001458	Coptisine	30.67185	0.85647
MOL002668	Worenine	45.83318	0.86552

DL = drug-likeness, OB = oral bioavailability.

#### 3.1.2. Acquisition of predicted targets of JHWD for PCOS-IR.

**Figure 2.**
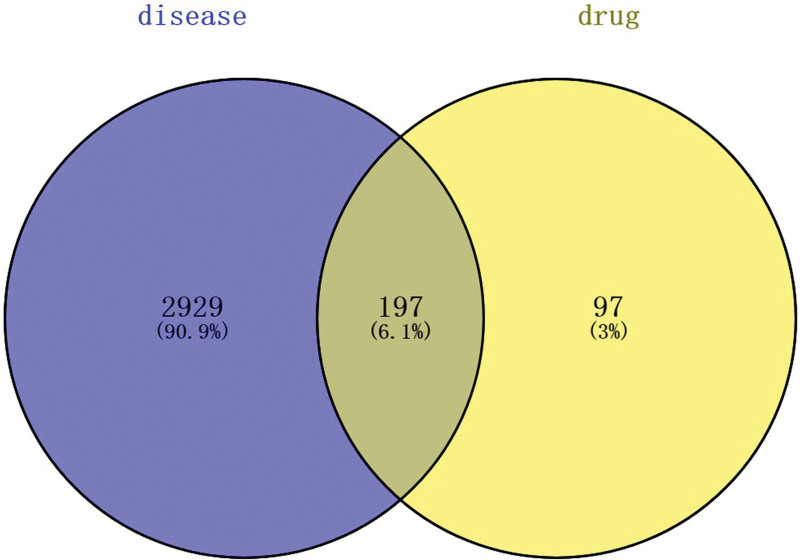
The overlap targets with Venny are the predicted key targets.

After eliminating duplicates, a total of 294 compound-related targets, as well as 3126 PCOS-related and IR-related targets, were retrieved from the databases mentioned above. These retrieved targets were then submitted into the Venny platform (https://bioinfogp.cnb.csic.es/tools/venny/) to acquire 197 key targets, as specified in Figure 2.

#### 3.1.3. Construction of key-target PPI and “drug-components-targets-disease” networks.

**Figure 3.**
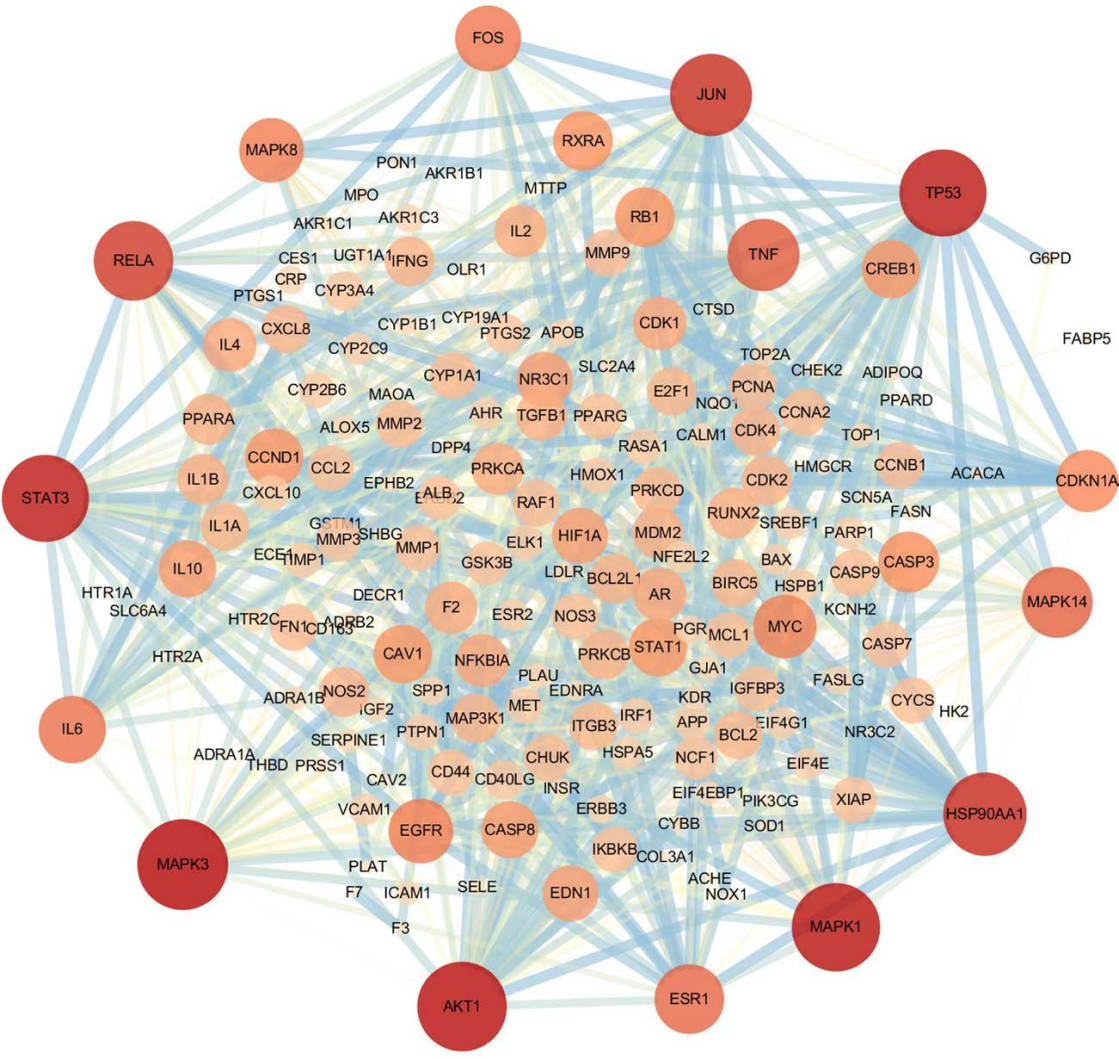
The key-target PPI network (circular nodes represent targets; the larger and darker nodes mean higher degree value; the thicker edges mean higher combination scores). PPI = protein-protein interaction.

**Figure 4.**
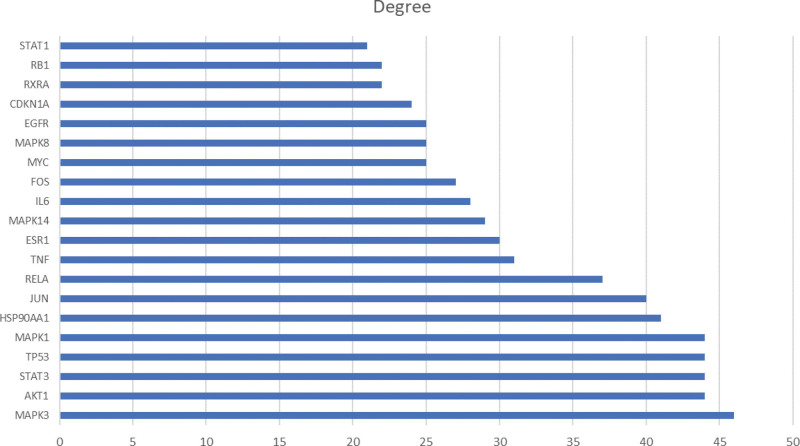
Degree ranking of top 20 key targets.

**Figure 5.**
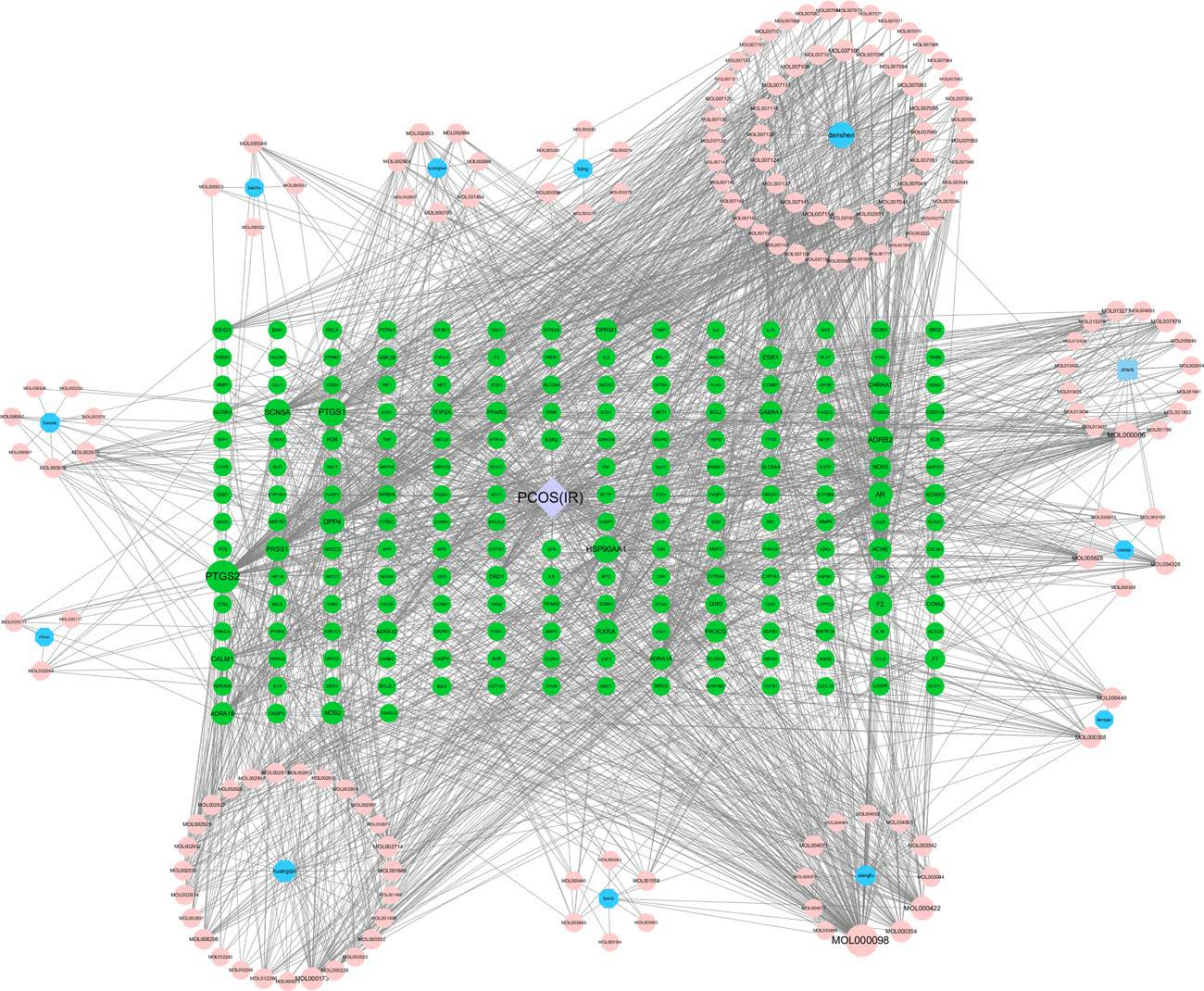
“Drug-Components-Targets-Disease” network (blue octagons represent herbs, pink ellipses represent active components, green ellipses substitute targets, and purple diamond represents the disease).

The 197 key targets were imported into the GeneMANIA database to construct the PPI network (Fig. 3). It should be noted that the lower confidence limit was set around 0.900, and the isolated nodes were eliminated. Moreover, the first 20 key targets were screened following the degree value (Fig. 4). The critical information of key targets, active components of JHWD, and their interrelation were imported into the Cytoscape 3.7.2 software for network visualization and analysis. Then, the “Drug-Components-Targets-Disease” network was constructed (Fig. 5). Notably, the network consisted of 361 nodes (12 herbs, 151 active components, 197 target nodes, and 1 disease node) and 2075 edges. The top 5 active components were selected following the degree value, as presented in Table 2.

**Table 2:** The top 5 active components by degree value.

Mol ID	Degree	Molecule name	Source
MOL000098	119	Quercetin	Xiangfu, Huanglian, Tusizi
MOL000006	52	Luteolin	Zhishi, Danshen, Xiangfu
MOL000422	49	Kaempferol	Xiangfu, Tusizi
MOL000173	39	Wogonin	Huangqin
MOL005828	31	Nobiletin	Zhishi, Chenpi

#### 3.1.4. GO and KEGG pathway enrichment analyses.

**Figure 6.**
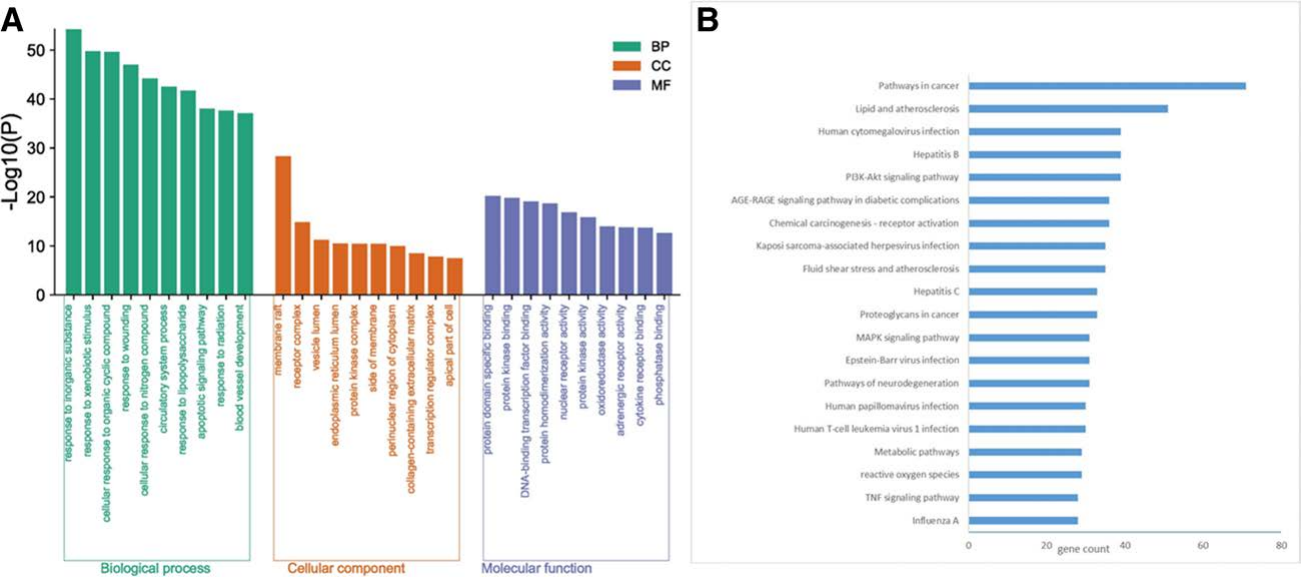
(A) GO enrichment analysis. (B) KEGG pathway analysis of 197 overlapping targets. GO = Gene Ontology, KEGG = Kyoto Encyclopedia of Genes and Genomes.

After obtaining 327 pathways, the disease-related targets were then incorporated into the KEGG database to demonstrate the underlying pathogenesis of PCOS with IR. As depicted in Table 3, the enriched pathways predominantly included cancer, metabolic, lipid and atherosclerosis, as well as phosphoinositide-3-kinase-protein kinase B (PI3K-Akt), and mitogen‐activated protein kinase (MAPK) signaling pathways. To elucidate the biological effects of JHWD for PCOS and IR, the resultant 197 key targets were inserted into the Metascape system for the GO enrichment analysis and the KEGG database for pathway analysis (Fig. 6).

**Table 3:** The top 5 enrichment pathways of PCOS with IR.

Pathway name	Numbers of targets
Pathways in cancer	197
Metabolic pathways	169
PI3K-Akt signaling pathway	144
Lipid and atherosclerosis	109
MAPK signaling pathway	103

IR = insulin resistance, MAPK = mitogen‐activated protein kinase, PCOS = polycystic ovary syndrome, PI3K-Akt = phosphoinositide-3-kinase-protein kinase B.

On the one end, the GO enrichment results were described as follows: Concerning the biological process (BP), these significant targets were predominantly related to various attributes of influence of inorganic substance, response to xenobiotic stimulus, lipopolysaccharide, radiation, organic, and nitrogen-based compounds, circulatory system process, apoptotic signaling pathway, as well as blood vessel development; In terms of cell composition, the main targets were associated with membrane raft, receptor complex, vesicle, and endoplasmic reticulum lumen, and protein kinase complex, among others; In terms of molecular functionalities, the targets were predominantly relevant to protein domain-specific binding, protein kinase-specific binding, deoxyribose nucleic acid (DNA)-binding transcription factor binding, and protein homo-dimerization as well as nuclear receptor activities, among others.

**Figure 7.**
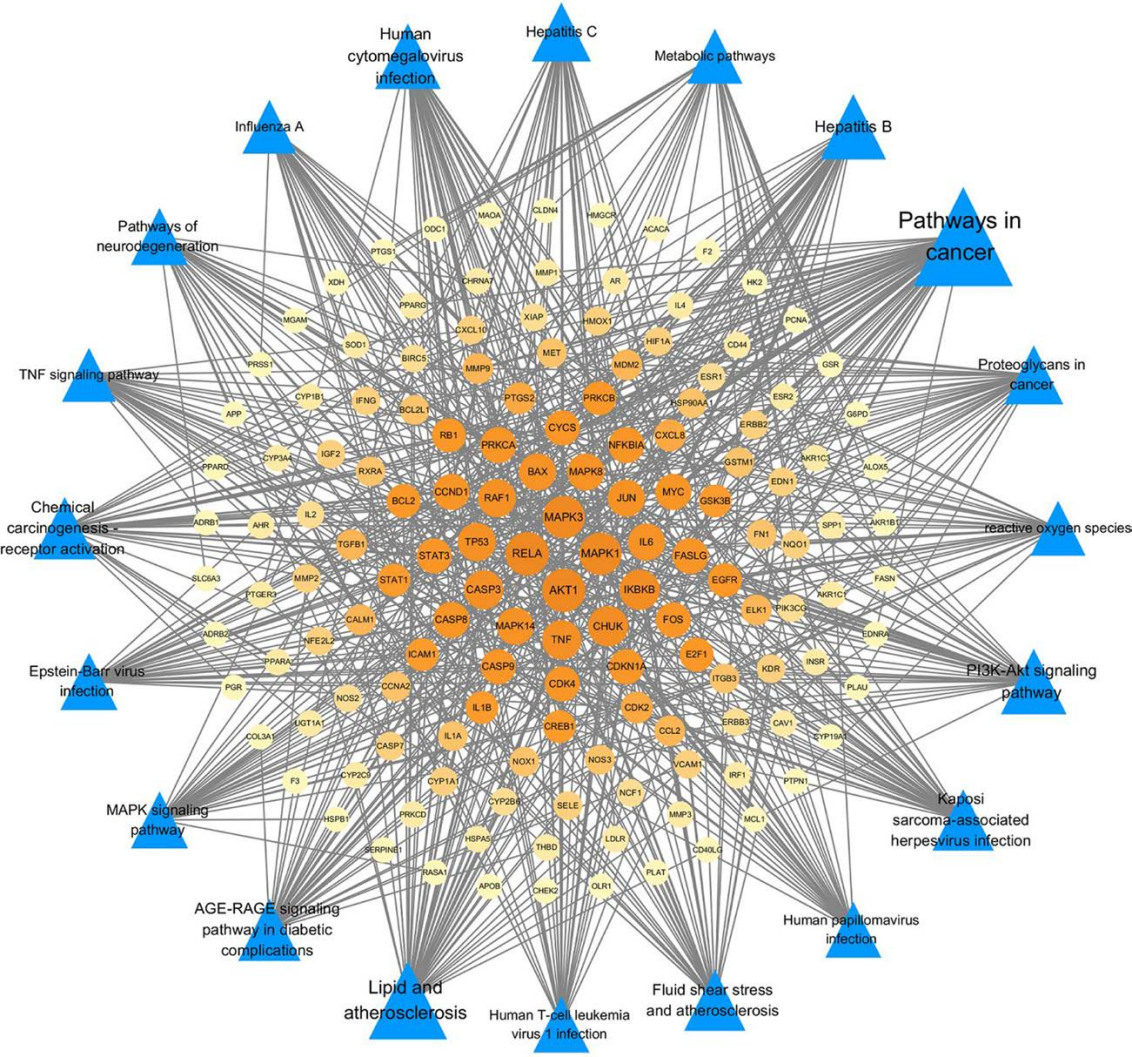
Pathway-target network. Blue triangle nodes represent the pathways. Yellow round nodes represent the key targets.

**Figure 8.**
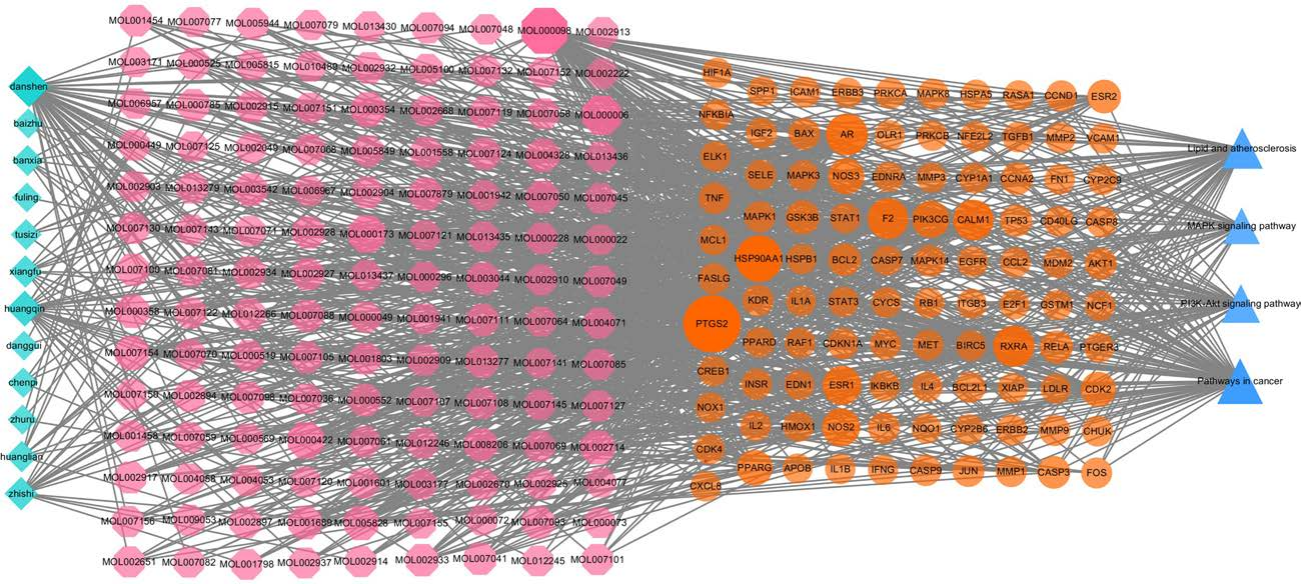
H-C-T-P (herb-component-target-pathway) network. Blue triangle nodes represent the pathways. Yellow round nodes represent targets. Pink octagon nodes represent components. Green diamond nodes represent herbs.

On the other end, the experimental results of KEGG pathway analysis demonstrated that the key targets of JHWD and PCOS-IR were predominantly associated with the pathways of cancer, lipid and atherosclerosis, PI3K-Akt signaling, hepatitis, human cytomegalovirus infection, advanced glycation endproducts-receptor for advanced glycation endproducts (AGE-RAGE) signaling in diabetic complications, chemical carcinogenesis-receptor activation, kaposi sarcoma-associated herpesvirus infection, chemical carcinogenesis-receptor activation, and MAPK signaling pathway, among others. The relationships between these notified main targets and pathways were submitted in the Cytoscape 3.7.2 software to establish the pathways-target network (Fig. 7). It was recognized from the literature that signaling pathways closely related to PCOS-IR included the pathways cancer, lipid and atherosclerosis, as well as PI3K-Akt, and MAPK signaling pathways. In addition, the H-C-T-P (herb-component-target-pathway) network was then built to define the interrelations of herbs, components, targets, and the first 4 pathways (Fig. 8). Further, it was observed that 71 proteins were enriched in pathways of cancer, 51 in the lipid and atherosclerosis, 39 in the PI3K-Akt signaling, and 31 in the MAPK signaling, contributing to the primary mechanisms of JHWD for PCOS with IR.

#### 3.1.5. Molecular docking between active components and key targets.

**Figure 9.**
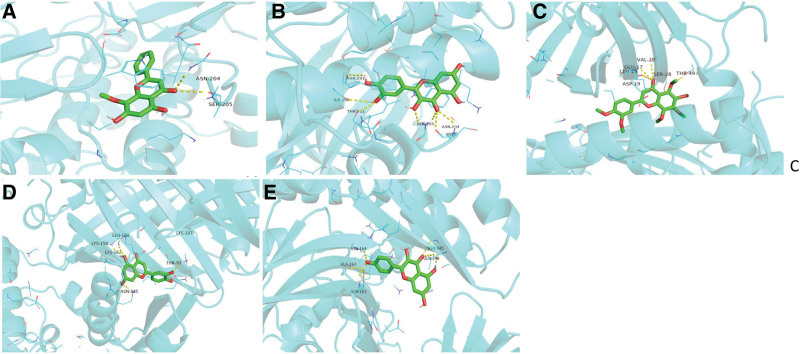
The molecular docking results of the interaction of active components with the key targets. (A) Binding pattern between wogonin and AKT1. (B) Binding pattern between quercetin and AKT1. (C) Binding pattern between nobiletin and TP53. (D) Binding pattern between luteolin and TP53. (E) Binding pattern between kaempferol and TP53. AKT1 = protein kinase B, TP53 = tumor protein 53, AKT1 = RAC-alpha serine/threonine-protein kinase.

The results of molecular docking showed that wogonin and quercetin docked well into RAC-alpha serine/threonine-protein kinase (AKT1), MAPK1, MAPK3, signal transducer and activator of transcription 3 (STAT3) and tumor protein 53 (TP53), while nobiletin, luteolin and kaempferol had strong binding affinity only with TP53. The results were shown in Table 4, and Figure 9 illustrated the strongest binding affinity of the active components for key target proteins.

**Table 4:** The binding free energy of top 5 active components with first 5 key targets.

Molecule name	Affinity (kcal/mol)				
	AKT1	MAPK1	MAPK3	STAT3	TP53
Wogonin	−9.6	−7.0	−6.5	−7.6	−6.5
Quercetin	−10.4	−6.6	−8.1	−6.9	−8.6
Nobiletin	0	0	0	0	−7.6
Luteolin	0	0	0	0	−8.5
Kaempferol	0	0	0	0	−8.6

AKT1 = RAC-alpha serine/threonine-protein kinase, MAPK = mitogen‐activated protein kinase, STAT3 = signal transducer and activator of transcription 3, TP53 = cellular tumor antigen p53.

### 3.2. Comparison of clinical efficacy

#### 3.2.1. Comparison of phlegm-dampness syndrome scores.

Before treatment, there were no significant differences in the TCM syndrome scores between the 2 groups. The phlegm-dampness syndrome of the experimental group showed significant improvement after treatment, compared with control group (Table 5).

**Table 5:** Comparison of phlegm-dampness syndrome score before and after treatment in the 2 groups.

Item	Experimental group		Control group	
	Baseline	Posttreatment	Baseline	Posttreatment
Syndrome score	21.79 ± 2.94	6.85 ± 1.12*#	21.46 ± 2.87	10.75 ± 1.63*

* *P* < .01, compared with baseline.

#*P* < .01, compared with the control group after treatment.

#### 3.2.2. Comparison of the rate of resumption of normal menstrual cycles and regular ovulation.

After treatment, 80% of patients of the experimental group regained normal menstrual cycles compared to 56.1% of the control group, and it was statistically significant (*P* < .05). There was significantly difference in the resumption of regular ovulation between the 2 groups (*P* < .05) (Table 6).

**Table 6:** The rate of resumption of normal menstrual cycles and regular ovulation of the 2 groups after treatment (n [%]).

Item	Experimental group	Control group
Normal menstrual cycles (n [%])	32 (80.0%)##	23 (56.1%)
Regular ovulation (n [%])	28 (70.0%)##	18 (43.9%)

##*P* < .05, compared with the control group after treatment.

#### 3.2.3. Lipids, glucose, insulin, and inflammation profiles.

The effects of JHWD on the levels of lipid (TC, TG, and LDL-C), glucose and insulin (FBG, FINS, and HOMA-IRI), and inflammatory cytokines (TNF-α, IL-6, and IL-1) in the blood samples of PCOS with IR patients were observed (Table 7). It should be noted that there existed no statistical differences in all indicators between the 2 groups before the treatment sample. In contrast, various indicators of the 2 groups were significantly decreased from baseline (*P* < .01) after therapy, in which these indicators of the experimental group were remarkably lower than those of the control group (*P* < .01). Together, it was evident that JHWD could regulate the levels of lipid, glucose, insulin, and inflammatory cytokines in the blood samples of PCOS with IR patients.

**Table 7:** TC, TG, LDL-C, FBG, FINS, HOMA-IRI, TNF-α, IL-6, IL-1, levels of the 2 groups.

Item	Experimental group		Control group	
	Baseline	Posttreatment	Baseline	Posttreatment
FBG (mmol·L^−1^)	5.81 ± 0.67	4.97 ± 0.43*#	5.78 ± 0.64	5.34 ± 0.48*
FINS (mU·L^−1^)	12.69 ± 2.34	8.34 ± 1.67*#	12.14 ± 2.21	10.05 ± 1.73*
HOMA-IRI	3.27 ± 0.51	1.85 ± 0.33*#	3.12 ± 0.47	2.37 ± 0.40*
TC (mmol·L^−1^)	5.28 ± 0.78	4.23 ± 0.54*#	5.16 ± 0.87	4.63 ± 0.54*
TG (mmol·L^−1^)	2.41 ± 0.81	1.66 ± 0.52*#	2.55 ± 0.54	2.01 ± 0.66*
LDL-C (mmol·L^−1^)	3.78 ± 0.31	3.22 ± 0.28*#	3.75 ± 0.33	3.32 ± 0.29*
TNF-α (ng·L^−1^)	32.78 ± 4.43	17.29 ± 2.38*#	33.16 ± 4.57	20.58 ± 2.69*
IL-6 (ng·L^−1^)	31.07 ± 4.36	17.53 ± 2.19*#	30.95 ± 4.47	21.19 ± 2.82*
IL-1 (ng·L^−1^)	28.12 ± 4.24	15.36 ± 2.25*#	27.74 ± 4.13	18.27 ± 2.50*

FBG = fasting blood glucose, FINS = fasting insulin, HOMA-IRI = homeostasis model assessment of insulin resistance index, IL-6 = interleukin-6, IL-1 = interleukin 1, LDL-C = low-density lipoprotein cholesterol, TC = total cholesterol, TG = triglycerides, TNF-α = tumor necrosis factor-alpha.

* *P* < .01, compared with baseline.

#*P* < .01, compared with the control group after treatment.

## 4. Discussion

In the current investigation, we are intended to reveal the possible mechanism of pathogenesis behind the PCOS with IR and the potential therapeutic effects and mechanistic exploration of JHWD through the network pharmacology approach along with preliminary validation through clinical experiments. In this context, a total of 197 overlapped targets and 20 predicted key targets may be the potential therapeutic targets for JHWD in the treatment of PCOS-IR. AKT1 has previously been shown to be localized in both granulosa cells and oocytes and involved in inflammation, cellular growth, survival and metabolism, and AKT1-deficient leads to reduced fertility.^[24]^ MAPK1 and MAPK3 play important roles in ovulation of PCOS involved in transmitting signals. It has been reported that the expression of MAPK1 and MAPK3 genotypes were differed significantly between PCOS patients and controls, and they were related to the levels of body mass index (BMI), HOMA-IR, and C-reactive protein (CRP).^[25]^ STAT3 activation is closely associated with PCOS-IR. It has been shown that the expression of phosphor-STAT3 and IL-6 were increased in the ovarian tissues of rat models of PCOS-IR,^[26]^ which suggested that STAT3 signaling pathway was activated mediated by IL-6 in the pathology of PCOS-IR. TP53 protein is mainly involved in promoting apoptosis and inhibiting proliferation. A previous study in India showed that there was a significant increase of TP53 genotype frequency in PCOS patients compared to controls,^[27]^ indicating that TP53 might contribute to genetic susceptibility to PCOS. Therefore, analysis of the predicted targets may represent the therapeutic targets for JHWD in the treatment of PCOS-IR.

To further identify the related signaling pathways of JHWD on PCOS-IR, the enrichment analyses of PCOS-IR-related targets were performed to confirm that the chief enriched pathways in the progress of PCOS with IR were related to the pathways in cancer, metabolic, PI3K-Akt signaling, lipid and atherosclerosis, as well as MAPK signaling, indicating their importance in the pathogenesis of PCOS with IR. Moreover, the abnormalities in insulin receptor and insulin-signaling pathways could contribute to the development of IR in PCOS patients, which could be indicated by the suppression of the PI3K-Akt signaling and the extreme instigation of the MAPK signaling pathways. In this context, several reports indicated that the dominant insulin pathways in regulating glucose and lipid metabolism included the PI3K-Akt and MAPK signaling pathways.^[28]^ Briefly, the PI3K-AKT signaling often regulates glucose and lipid metabolism.

In contrast, the inhibition of PI3K-AKT signaling can block the proliferation efficacy of various cancer cells, promoting their apoptosis and eventually death.^[29,30]^ In addition, the PI3K-Akt/NF-κB pathway is an important signaling pathway in the chronic low-grade inflammation of PCOS.^[31]^ In this case, it was demonstrated that blocking the MAPK signaling pathway could substantially suppress the proliferation of KGN cells (human ovarian granulosa cell line) of PCOS.^[32]^ Lipid and atherosclerosis are the metabolic, inflammatory reactions that may contribute to dysfunction of the vascular endothelial cells and lipid metabolism disorder of PCOS with IR.^[33]^ In this framework, several reports demonstrated that the generated inflammatory response was the leading contributor to the pathogenesis of IR in women with PCOS conditions.^[34]^ Thus, a chronic inflammation-mediated metabolic dysfunction IR might be the underlying pathogenesis of PCOS, which could be demonstrated by relying on these crucial pathways.

In this study, it was observed that the analysis of the active compounds of JHWD resulted in several notable active compounds, such as quercetin, luteolin, kaempferol, wogonin, and nobiletin, among others. Moreover, the primary enriched pathways included cancer, lipid and atherosclerosis, PI3K-Akt and MAPK signaling pathways, advanced glycation endproducts-receptor for advanced glycation endproducts (AGE-RAGE) signaling in diabetes-associated complications, human cytomegalovirus, and hepatitis B, confirmed by enrichment analysis of overlapping targets of JHWD and PCOS-IR. These pathways were closely correlated with multiple biologic processes, including response to hormone and inorganic substance, as well as a cellular response to lipid, cytokine, and xenobiotic stimulus, positive regulation of cell migration, as well as inflammatory responses. Previous studies indicated that quercetin could substantially increase insulin sensitivity and alleviate IR by acting on the MAPK signaling pathway.^[35]^ In a case, quercetin exerted anti-inflammatory effects by inhibiting the NF-κB and MAPK signaling pathways.^[36]^ In another case, luteolin significantly reduced the fasting insulin and blood glucose, as well as serum lipid levels in hyperlipidemic rats.^[37]^ Moreover, quercetin and luteolin synergistically decreased the generation of inflammatory cytokines (TNF-α, IL-6, and IL-1β).^[38]^

Interestingly, it was observed that kaempferol could ameliorate atherosclerosis significantly through lowering lipids and alleviating vascular inflammation.^[39]^ In another case, it was demonstrated that kaempferol could play an anti-inflammatory role via the NF-κB and MAPK pathways.^[40]^ Wogonin suppressed IL-6, TNF-α, and (cyclooxygenase-2) COX-2 production in peritoneal macrophages^[41]^ induced apoptosis by modulating the PI3K-AKT pathway.^[42]^ Consequently, based on the above network pharmacology approach and previous literature reports, it could be concluded that the participation of active components of JHWD in the regulation of lipid and atherosclerosis, PI3K-Akt, and MAPK signaling pathways might be the underlying mechanisms of JHWD for PCOS-IR.

Meanwhile, the molecular docking analysis was carried out on the key targets to improve the accuracy of network pharmacological prediction. The docking result of the 5 active compounds and the 5 key target proteins showed that wogonin and quercetin had good affinity with AKT1, MAPK1, MAPK3, STAT3, and TP53, while nobiletin, luteolin and kaempferol had strong binding affinity with TP53.

Together, these results strongly suggested that the main active components in JHWD could effectively treat PCOS-IR by acting on these key target proteins, involving multiple signaling pathways. Moreover, the clinical validation of this study explored that JHWD exhibited significant clinical efficacy in improving the clinical syndromes, menstrual cyclicity and ovulatory function, and remarkably reduced the lipid levels (TC, TG, and LDL-C), the glucose and insulin levels (FBG, FINS, and HOMA- IRI), and the inflammatory cytokines (TNF-α, IL-6, and IL-1β) in the blood samples of PCOS with IR patients. Therefore, JHWD showed exceptional therapeutic events towards regulating the glycolipid metabolism, reducing IR, and exerting anti-inflammatory effects.

## 5. Conclusion

In summary, we employed the network pharmacology approach, molecular docking analysis and a clinical study to demonstrate the significant clinical potential of multiple components in JHWD for the treatment of PCOS-IR. It was observed that a chronic inflammation-mediated metabolic dysfunction predominantly associated with lipid and atherosclerosis, PI3K-Akt, and MAPK signaling pathways might be the underlying pathogenesis of PCOS with IR. However, JHWD dramatically exerted functions of anti-inflammatory and improved glycolipid metabolism. We firmly believe that JHWD will offer a potential value to exploring the pathogenesis of PCOS and the promotion of Chinese classical prescriptions, indicating their ability towards eradicating various metabolic diseases.

## Author contributions

NS designed the study, analyzed the data, and wrote the article. YZ collected and analyzed parts of the data. HM supervised the study and revised the article. All authors read the final version and approved submission.

**Conceptualization:** Na Shi.

**Data curation:** Yuhe Zhou.

**Formal analysis:** Na Shi.

**Funding acquisition:** Hongbo Ma.

**Investigation:** Na Shi.

**Methodology:** Na Shi.

**Project administration:** Na Shi.

**Resources:** Na Shi, Yuhe Zhou.

**Software:** Na Shi.

**Supervision:** Hongbo Ma.

**Validation:** Na Shi.

**Visualization:** Na Shi.

**Writing – original draft:** Na Shi.

**Writing – review & editing:** Na Shi, Hongbo Ma.
